# Resistome Analysis of *Klebsiella pneumoniae* Complex from Residential Aged Care Facilities Demonstrates Intra-facility Clonal Spread of Multidrug-Resistant Isolates

**DOI:** 10.3390/microorganisms12040751

**Published:** 2024-04-08

**Authors:** Jack M. Blaikie, Sylvia A. Sapula, Naomi L. Siderius, Bradley J. Hart, Anteneh Amsalu, Lex E.X. Leong, Morgyn S. Warner, Henrietta Venter

**Affiliations:** 1 UniSA Clinical and Health Sciences, Health and Biomedical Innovation, University of South Australia, Adelaide, SA 5000, Australia; jack.blaikie@mymail.unisa.edu.au (J.M.B.); sylvia.sapula@unisa.edu.au (S.A.S.); sidnl002@mymail.unisa.edu.au (N.L.S.); brad.hart@unisa.edu.au (B.J.H.); anteneh.geremew@unisa.edu.au (A.A.); lex.leong@sa.gov.au (L.E.X.L.); 2 Department of Medical Microbiology, University of Gondar, Gondar 196, Ethiopia; 3 Microbiology and Infectious Diseases, SA Pathology, Adelaide, SA 5000, Australia; morgyn.warner@sa.gov.au; 4 School of Medicine, University of Adelaide, Adelaide, SA 5000, Australia; 5 Infectious Diseases Unit, Royal Adelaide Hospital, Adelaide, SA 5000, Australia

**Keywords:** antimicrobial resistance, multidrug resistance, biocide resistance, clonal dissemination, wastewater-based epidemiology, ceftazidime, ciprofloxacin

## Abstract

Antimicrobial-resistant *Klebsiella pneumoniae* is one of the predominant pathogens in healthcare settings. However, the prevalence and resistome of this organism within residential aged care facilities (RACFs), which are potential hotspots for antimicrobial resistance, remain unexplored. Here, we provide a phenotypic and molecular characterization of antimicrobial-resistant *K. pneumoniae* isolated from RACFs. *K. pneumoniae* was isolated from urine, faecal and wastewater samples and facility swabs. The antimicrobial susceptibility profiles of all the isolates were determined and the genomic basis for resistance was explored with whole-genome sequencing on a subset of isolates. A total of 147 *K. pneumoniae* were isolated, displaying resistance against multiple antimicrobials. Genotypic analysis revealed the presence of beta-lactamases and the ciprofloxacin-resistance determinant QnrB4 but failed to confirm the basis for the observed cephalosporin resistance. Clonal spread of the multidrug-resistant, widely disseminated sequence types 323 and 661 was observed. This study was the first to examine the resistome of *K. pneumoniae* isolates from RACFs and demonstrated a complexity between genotypic and phenotypic antimicrobial resistance. The intra-facility dissemination and persistence of multidrug-resistant clones is concerning, given that residents are particularly vulnerable to antimicrobial resistant infections, and it highlights the need for continued surveillance and interventions to reduce the risk of outbreaks.

## 1. Introduction

Antimicrobial resistance (AMR) is an accelerating global emergency [1]. AMR is annually associated with 4.95 million deaths, and directly causes 1.27 million deaths globally—which is estimated to be more than HIV/AIDS or malaria [2]. Without more effective intervention, AMR may become the single largest cause of global mortality by 2050 [3]. Species of the *Klebsiella pneumoniae* complex, which includes *K. variicola* [4], are amongst the most prevalent global AMR pathogens, responsible for 193,000 annual deaths while contributing to an additional 600,000 [2]. Within Australia alone, *K. pneumoniae* complex bloodstream infections have an associated 13.4% 30-day all-cause mortality [5]. *K. pneumoniae* complex isolates are intrinsically resistant to many antimicrobials, and they are increasingly resistant to the antimicrobials relied on as treatment options, such as cephalosporins, fluroquinolones and carbapenems [5]. Carbapenem-resistant and third-generation cephalosporin-resistant *K. pneumoniae* each currently cause between 50,000 and 100,000 deaths annually [2]. Additionally, fluroquinolone resistance, often plasmid-mediated, is increasingly identified in clinical isolates, further complicating potential treatment regimens [6,7].

The genome of *K. pneumoniae* is highly variable, with more than half comprising accessory genes [8,9,10]. This genomic plasticity facilitates *Klebsiella* spp. both acquiring and disseminating resistance genes, via plasmids and other mobile genetic elements, which are responsible for much of the observed resistance [8,9,10]. Both intrinsic and acquired resistance mechanisms within *K. pneumoniae* have been studied extensively, with the presence of resistance determinants correlating to a resistant phenotype [8,10,11]. However, emerging, and inexplicable antimicrobial resistance within *Klebsiella* spp. has been identified [12,13,14,15].

A known contributor in the development of AMR is the frequent use of antibiotics, which in residential aged care facilities (RACFs) is well documented [16,17]. Residents of RACFs are older, frequently immunocompromised and prone to bacterial infections necessitating antimicrobial treatment. There is well-documented inappropriate extended use of antimicrobial prophylaxis in this population [18]. As in many institutions, there is also a high use of biocidal cleaning agents [16,19,20]. Additionally, close living conditions and frequent transfers of residents to other healthcare settings could promote AMR development and dissemination [21,22,23]. Current surveillance characterizes RACFs as significant reservoirs of antimicrobial-resistant organisms [16,24,25,26,27], with rates of AMR similar to those in hospitals [5]. Reported outbreaks of drug-resistant *K. pneumoniae* from long-term care facilities indicate a potential presence of AMR *K. pneumoniae* within RACFs [28,29]. However, no study has yet assessed the prevalence nor the resistome and antimicrobial susceptibility profiles of the WHO priority pathogen *K. pneumoniae* complex isolates within this setting.

This study reports on the surveillance of *K. pneumoniae* complex collected from RACFs in Adelaide, Australia. The comprehensive sampling regime included residents, the facility and the wastewater generated by the facility. The phenotypic antimicrobial resistance profiles of isolates were characterized and compared with genotypic analyses of resistance determinants. This pilot surveillance study aimed to provide, to our knowledge, the first comprehensive report on the resistome of *Klebsiella pneumoniae* complex isolates from RACFs and on the prevalence of resistant *K. pneumoniae* in aged care facilities, and it contributes to a more comprehensive understanding of resistance determinants within this species.

## 2. Materials and Methods

### 2.1. Sampling

Faecal, urine, facility and wastewater samples were collected from within aged care facilities (‘Facility 1’, 170 beds; ‘Facility 2’, 70 beds; ‘Facility 3’, 58 beds) and a retirement cohort (‘Retirement’, 38 beds total) in Adelaide, Australia. Faecal and urine samples were collected by a registered nurse.

For the collection of facility environmental swabs, which was performed across all facilities, sterile cotton-tipped applicators moistened in sterile saline (0.9% *w*/*v*) were wiped over touchpoints, and bathroom sinks, including drains, within each residence. Swabs were transported to the laboratory on ice, re-suspended in 200 µL peptone water with 20% *v*/*v* glycerol as cryoprotectant and stored at −80 °C until processing.

Wastewater sampling was performed at 3-month intervals from October 2019 to February 2021 and was not performed at Facility 3 due to physical lack of access. In each instance, wastewater samples of ~200 mL were collected hourly, for ten hours, and pooled for analysis.

All samples were collected on-site and held at 4 °C during transit to the host laboratory immediately after collection for same-day processing.

### 2.2. Isolation and Identification of K. pneumoniae

Faecal, facility and wastewater samples were plated onto rehydrated Brilliance™ *E. coli*/coliform selective agar (dehydrated, CM1046B, Thermo Scientific™, Melbourne, Australia) supplemented with either 1 mg/L ceftazidime (Sigma, Melbourne, Australia), 0.1 mg/L ciprofloxacin (Sigma, Australia) or no supplementation, whilst urine samples were plated onto rehydrated Brilliance™ UTI agar (dehydrated, CM0949B, Thermo Scientific™, Australia). Non-selective rehydrated Columbia agar base (dehydrated, NCM0038A, Neogen Australia, Bundamba, Australia) was used throughout the study to assess bacterial growth. Plates containing wastewater and facility samples were incubated at 25 °C for 48 h, whilst faecal and urine plates were incubated at 37 °C for 24 h.

Following colony purification, morphologically distinct colonies were identified to species and subspecies by matrix-assisted laser desorption/ionization time-of-flight mass spectrometry (MALDI-TOF) (Bruker Daltonik GmbH, Bremen, Germany). *Klebsiella* spp. were stored in cryoprotective enrichment media, tryptic soy broth (dehydrated, CM0129, Thermo Scientific™, Australia) + 20% *v*/*v* glycerol, at −80 °C.

### 2.3. Antimicrobial Susceptibility Testing

*K. pneumoniae* complex isolates were subjected to antimicrobial susceptibility assays using the broth microdilution method as outlined in the European Committee on Antimicrobial Susceptibility Testing (EUCAST) [30] guidelines. ‘Resistance’ was defined as a minimum inhibitory concentration (MIC) above the EUCAST breakpoint value [30]. Given the increasingly published links between antimicrobial resistance and biocide tolerance [31,32], tolerance to the biocides benzalkonium chloride, chlorhexidine and triclosan was also assessed using the same method and was determined by comparison with reported ECOFF values for *K. pneumoniae* [33]. *Escherichia coli* ATCC 25922 was used as a quality control throughout.

### 2.4. Efflux Pump Inhibition Assays

Assays quantifying the synergism between antimicrobials and efflux pump inhibitors were performed in accordance with methods widely described within the literature [32,34,35,36]. Bacterial growth was assessed in the presence of antimicrobials in combination with the RND-family efflux pump inhibitor phenylalanine-arginine β-naphthylamide (PAβN), to investigate if active efflux was responsible for the observed antimicrobial resistance.

### 2.5. Genomic DNA Isolation and Whole-Genome Sequencing

For whole-genome sequencing (WGS), DNA was extracted using an MN NucleoSpin^®^Microbial DNA kit (Machery-Nagel GmbH and Co.KG, Duren, Germany), following the manufacturer’s instructions. The quantity (ng/μL) and purity (absorbance 260 nm:280 nm) of DNA was assessed using a Cytation5 imaging reader (BioTek instruments, Winoosi, VT, USA). Extracted genomic DNA was visualised on 1% w/v agarose gel via electrophoresis to ensure no shearing of extracts. WGS was performed at SA Pathology Adelaide, Australia. The Nextera XT DNA library preparation kit (Illumina Inc., San Diego, CA, USA) was used to prepare sequenced libraries, as per the manufacturer’s instructions. Sequencing was performed on the Illumina NextSeq 550 platform with the NextSq 500/550 Mid-Output kit v2.5 for 300 cycles (Illumina Inc.).

### 2.6. Bioinformatic and Statistical Analyses

Raw paired-end sequencing reads were assembled and annotated using the TORMES pipeline version 1.3 [37]. Following the removal of sequence adaptors by Trimmomatic version 0.4 [38], sequence quality assessment was performed by Prinseq version 0.20.4 [39] and Sickle version 1.33 [40]. Draft genomes were then assembled by SPAdes version 3.15.4 [41] and annotated by Prokka version 1.14.5 [42]. Draft genomes were screened for antimicrobial resistance genes using ResFinder version 2.1 [43], the Antibiotic Resistance Gene-ANNOTation (ARG-ANNOT) database [44] and the comprehensive antibiotic resistance database (CARD) [45].

Identification of single-nucleotide polymorphisms (SNPs) within whole genomes was performed using CSIphylogeny V1.4 [46], in August 2023, with parameters as follows: minimum depth at SNP position at 10×, minimum distance between SNPs at 10 bp and minimum SNP quality score of 30. Isolates were classified as clones based on previous research into *K. pneumoniae* clonality and sample collection information [47,48,49]. Plasmid presence and replicons were assessed using PlasmidFinder 2.1 [50].

Downstream visualisation and annotation of phylogenetic data, which were generated within the TORMES pipeline, was achieved through the use of the Interactive Tree of Life v.6 (iTOL) [51]. Bar graphs were generated using GraphPad Prism v.9 [52]. Manual probing of nucleotide and amino acid sequences of key genes and proteins was performed by sequence aligning tools Clustal omega [53] and ESPript v3.0 [54] to identify the presence of mutations.

### 2.7. Data Availability

The collective WGSs of isolates used in this study were deposited to the NCBI database BioProject ‘PRJNA949397′ (Table 1).

### 2.8. Ethics Approval

Ethics approval to collect clinical samples, which included faeces and urine, from RACF residents was granted by the University of South Australia’s human research ethics committee (Application ID: 201882).

## 3. Results

### 3.1. A Total of 147 K. pneumoniae Complex Isolates Were Identified

Faecal, urine and facility swabs were collected from 123 residents across three RACFs and from a retirement living cohort in Adelaide, Australia, while the wastewater from the facilities was also sampled as representative of all the residents and to capture clonal dissemination. A total of 147 *K. pneumoniae* complex isolates were identified from these samples (Table 2). Isolates were predominantly recovered from faecal samples, with 56% (n = 82) of isolates from this source. The 82 faecal and 2 urine *K. pneumoniae* complex isolates are representative of 40 participants, as some participants provided multiple samples. The next largest source was wastewater, with 40% (n = 59) of isolates derived from wastewater samples. Facility swabs yielded n = 4 (2%) of isolates, and urine samples yielded n = 2 (1%) of isolates.

At the species level, 134 (91%) of *K. pneumoniae* complex isolates were *K. pneumoniae* and 13 (9%) were *K. variicola*. The number of isolates recovered was proportionate to the number of beds in each facility, with Facility 1 having both the greatest number of beds (n = 170) and greatest number of isolates (n = 97, 66 %).

### 3.2. K. pneumoniae Complex Resistant to Ceftazidime, Ciprofloxacin and Trimethoprim-Sulfamethoxazole was Isolated

Since ESBL producing Enterobacterales have been highlighted as a problem in RACFs in Australia [55,56], the residents were screened for carriage of ESBL producing *K. pneumoniae* using Brilliance™ *E. coli*/coliform selective agar supplemented with 1 mg/L ceftazidime. *K. pneumoniae* were isolated from 7 of the 102 participants from the RACFs using this media, relating to a 7% incidence of colonization, which is similar to the national average and correlates with a previous study on the incidence of other Enterobacterales in RACFs [56]. However, when the antimicrobial susceptibility of all the isolates was determined, incidence of ceftazidime and cefepime resistance was much higher at 46% and 16%, respectively (Table 3), most likely due to the clonal spread of ceftazidime-resistant *K. pneumoniae* that were also isolated from wastewater and the facility. Most of the cephalosporin-resistant isolates displayed high-level ceftazidime resistance with MIC values of >64 mg/L, values well above the resistance breakpoint of 4 mg/L. Only 11% of the ceftazidime isolates were from agar plates supplemented with ceftazidime, indicating that the ceftazidime resistance observed was not merely due to selection bias during isolation.

Similarly, a high incidence of resistance was observed against ciprofloxacin, with 69% (n = 102) of isolates being ciprofloxacin-resistant (MIC ≥ 0.5 mg/L), with only 15% of the ciprofloxacin-resistant isolates being isolated from plates containing ciprofloxacin. Further, high-level ciprofloxacin resistance (MIC ≥ 64 mg/L) was observed for 5% (n = 8) of isolates. The incidence of ciprofloxacin resistance was followed by trimethoprim-sulfamethoxazole resistance, which was observed in 47% (n = 69) of isolates.

Carbapenem antimicrobials, primarily meropenem, are currently treatments relied on for AMR *K. pneumoniae* [5]. No isolates were resistant to the carbapenem meropenem; however, 3% (n = 4) of isolates were non-wild type with MIC values 1-2-fold above EUCAST breakpoints. One of the isolates was resistant to the last resort antibiotic colistin with an MIC of 32 µg/mL.

Overall, aminoglycoside resistance was relatively low compared to the instance of resistance to other antibiotics tested, with tobramycin and amikacin resistance observed in 10% (n = 15) and 5% (n = 8) of isolates, respectively.

Given the link between biocide tolerance and antimicrobial resistance [31,32], and the risk of background low levels of biocides causing AMR [53,54], the biocide sensitivity of isolates was also investigated (Table 4). No EUCAST breakpoints and ECOFF data are available for biocides. Therefore, our data were compared to published ECOFF values [33] to infer tolerance to biocides (Table 4). Three commonly used biocides were assessed in this study, namely, triclosan, chlorhexidine digluconate and benzalkonium chloride, all of which have been linked to the development of AMR [57,58]. Almost no tolerance to chlorhexidine was observed, with only 1.4% (n = 2) of isolates displaying an MIC above the ECOFF threshold of 64 mg/L. This contrasts with the tolerance to triclosan that was observed for a relatively high proportion (n = 67, 46%) of isolates. Tolerance to benzalkonium chloride was observed for n = 119 (96%) of isolates.

### 3.3. Multidrug Resistance (MDR) Was Prevalent within RACFs, Especially within Facility 1

**Figure 1:**
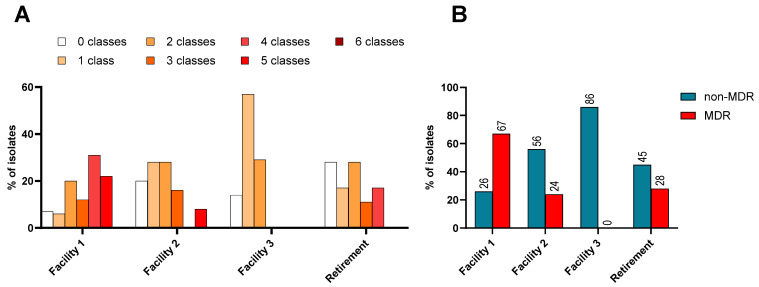
(**A**): Percentage of isolates resistant to 0–6 different classes of antimicrobials, grouped by number of classes and differentiated by facility. (**B**): The distribution of non-MDR and MDR *K. pneumoniae* complex isolates from across the three RACFs and one retirement setting within this study.

Each facility harboured some isolates which were sensitive to all classes of antimicrobials, but most isolates (n = 115, 78%) displayed resistance to one or more classes of antimicrobial tested and were thus AMR organisms (Figure 1A). Of the 40 residents who provided faecal and urine samples, n = 35 (87.5%) were found to be carrying an AMR organism.

The problem of *K. pneumoniae* complex is amplified when organisms display multidrug resistance (MDR), as treatment options are fewer. An MDR organism is defined as displaying resistance to antimicrobials in three or more different classes. Facility 1 had the highest proportion of MDR isolates at 72% (Figure 1B). This facility was also the only facility with isolates that were resistant to five classes of antibiotics tested and it had the lowest proportion of isolates without any resistance, which was 7% (Figure 1A).

### 3.4. Resistome Analysis of a Subset of K. pneumoniae Complex Could Not Identify the Genomic Basis for the Cephalosporin or Colistin Resistance

With antimicrobial susceptibility testing revealing a broad range of antimicrobial resistance phenotypes within the RACF *K. pneumoniae* complex isolates, whole-genome sequencing (WGS) was performed to elucidate the genetic bases of this resistance. A cohort of the 147 isolates were selected for sequencing, based on their resistance profiles (Table 5). Isolates displaying resistance to ceftazidime, cefepime and ciprofloxacin were targeted for WGS as relatively a high incidence of resistance was observed against these antimicrobials. Isolates both resistant and sensitive to the last-resort treatment option colistin were also included [59,60]. Additionally, an isolate sensitive to all the antibiotics tested (A031) was also included to allow for comparisons. All isolates selected were from Facility 1, the site with the highest proportions of MDR organisms observed.

**Figure 2:**
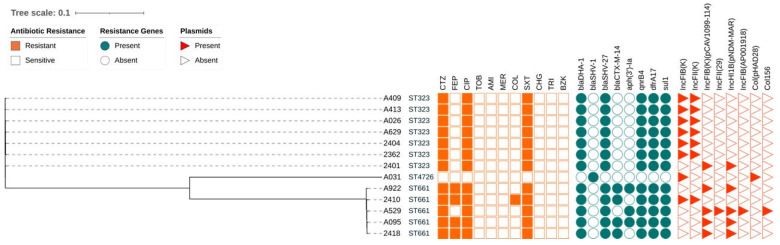
Phylogenetic tree constructed from core-genome SNPs of *K. pneumoniae* complex isolates. Sequence types, antimicrobial susceptibility (orange squares), presence of resistance determinants (teal circles) and plasmid presence (red triangles) are annotated to the phylogenetic tree. Presence is indicated by a shaded field, absence indicated by an empty field. CTZ: ceftazidime; FEP: cefepime; CIP: ciprofloxacin; TOB: tobramycin; AMI: amikacin; MER: meropenem; COL: colistin; SXT: trimethoprim-sulfamethoxazole; CHG: chlorhexidine digluconate; TRI: triclosan; BZK: benzalkonium chloride.

Resistome analysis revealed the presence of four beta-lactam-resistance determinants, *bla*_DHA-1_, *bla*_SHV-1_, *bla*_SHV-27_ and *bla*_CTX-M-14_, while no carbapenemases were identified (Figure 2). Isolate A031 was the only isolate carrying *bla*_SHV-1_, a cephalosporin-hydrolysing beta-lactamase which does not hydrolyse oxyimino cephalosporins such as ceftazidime [61]. Accordingly, isolate A031 was sensitive to ceftazidime. The other twelve sequenced isolates contained the AmpC beta-lactamase *bla*_DHA-1_ and a variant of *bla*_SHV-1_, *bla*_SHV-27_ and were resistant to ceftazidime. Previous studies have shown that both *bla*_DHA-1_ and *bla*_SHV-27_ can confer resistance to third-generation cephalosporins such as ceftazidime [61,62,63,64].

Four isolates were resistant to the fourth-generation cephalosporin cefepime. The determinant for the cefepime resistance was not identified.

The *bla*_SHV-27_ gene is frequently associated with other ESBL genes in resistant organisms, such as the CTX-M-type [65,66], consistent with findings here. Four of the sequenced isolates with differing cephalosporin resistance phenotypes co-harboured both the *bla*_SHV-27_ gene and a *bla*_CTX-M-14_: isolates A922, 2410, A095 and 2418. The beta-lactamase *bla*_CTX-M-14_ is part of the CTX-M class A ESBLs, which also include CTX-M-15 [67]. Unlike CTX-M-15, which is highly active against cefotaxime and is shown to be increasing in its ability to hydrolyse ceftazidime [68,69], CTX-M-14 is not typically associated with ceftazidime or cefepime resistance [70]. Therefore, despite isolates A922, 2410, A095 and 2418 harbouring this gene and displaying a resistant phenotype, *bla*_CTX-M-14_ might not be the resistance determinant responsible for cefepime resistance. The results obtained and the resistance determinants identified in this study do not clearly explain the high levels of ceftazidime resistance detected in many of the *K. pneumoniae* isolates assessed here. In addition, none of these beta-lactamases are known to confer cefepime resistance, which is observed in isolates A922, A095, 2410 and 2418. The presence of the identified beta-lactamases *bla*_SHV-1_, *bla*_SHV-27_ and *bla*_CTX-M-14_ therefore does not correlate conclusively with the resistance phenotypes observed against the beta-lactam antimicrobials assessed in this study.

Fluoroquinolone resistance was assessed using ciprofloxacin, and widespread ciprofloxacin resistance was observed. The fluoroquinolone-resistance determinant QnrB4, an alternate target protein which binds to gyrase enzymes, thus offering protection to host organisms against the quinolone class drugs, was identified and correlated largely with the observed ciprofloxacin resistance here (Figure 2) [71]. Typically, carriage of the *qnrB4* gene in isolation is correlated with only low-level resistance to ciprofloxacin [66,67], as was observed for the isolates that were sequenced in this study.

The observed trimethoprim-sulfamethoxazole resistance could be attributed to the presence of known resistance determinants. Trimethoprim-sulfamethoxazole-resistance determinants *dfrA17* and *sul1* (an integron-encoded dihydrofolate reductase and dihydropteroate synthase [72,73], respectively) were identified, and they were present in all isolates with a resistant phenotype (Figure 2).

Finally, colistin resistance was observed for isolate 2410 (Table 5); however, the colistin mobile resistance gene mcr and alleles were not present in any isolate (Figure 2).

### 3.5. Mutations in Genes Known to Confer Resistance to Antimicrobials

Despite the observed phenotypic resistance to cephalosporins and colistin, no well-known genetic resistance determinants were present, and those determinants that were found did not entirely correlate with resistance. Similarly, no genetic determinants were identified for the high-level resistance to ciprofloxacin observed for two of the isolates. Therefore, a manual probing for mutations in specific genes, which when mutated are known to confer resistance to antimicrobials, was performed across the sequenced isolates to identify mutations which could be responsible for the resistant phenotypes (Supplementary Table S1).

No mutations in the fluoroquinolone target proteins GyrA/B or ParE were observed in isolates with a ciprofloxacin-resistant phenotype. A ParC M304S substitution mutation was observed in six ciprofloxacin-resistant isolates but was absent in the other ciprofloxacin-resistant isolates, making the deduction of its role in ciprofloxacin resistance difficult to determine. Further, this mutation did not correlate with the ciprofloxacin MIC values observed. This mutation has also not been reported for other ciprofloxacin-resistant bacteria and needs further study to clarify its role.

Resistance to colistin within *Klebsiella* has been reported to be conferred by the presence of mutations in the *phoP/Q*, *mgrB*, *eptA* and *eptB* genes [67,68,69,70,71]. Therefore, these genes were probed for mutations. No mutations were observed in the transcriptional regulatory proteins PhoP and PhoQ for any of the sequenced isolates. The sole colistin resistance isolate, 2410, contained an M66I mutation in PrmA, T240M and T246A, mutations in PmrB and a T224M mutation in EptA. However, mutations were also present in the colistin-sensitive isolates A095, A529, 2418 and A922; hence, it could not be responsible for the observed colistin resistance. Both the PmrA and PmrB mutations identified in this study have also been determined in colistin-resistant *K. pneumonia* isolates from China [74]; however, their role in colistin resistance has not been experimentally verified. No mutations within the PhoPQ-negative regulator MgrB were observed.

In addition to probing for mutations which could confer colistin resistance, mutations which could account for the observed resistance to ceftazidime and cefepime were investigated in the sequenced isolates. The amino acid sequences of the *bla*_CTX-M-14_ beta-lactamases present in each cefepime-resistant isolate but also a cefepime-sensitive isolate were probed for mutations, as mutations here have been recognized as affecting the substrate specificity [75], but none were identified. Mutations in outer membrane porins OmpK35, 36 and 37 are known to correlate with resistance to some beta-lactam antibiotics (cephalosporin and carbapenem resistance) for *K. pneumoniae* [15,76,77,78,79,80,81]. As all sequenced isolates apart from A922 were resistant to ceftazidime, and some to cefepime, these porins were also examined for mutations which could affect porin function. Twelve of the thirteen isolates contained a K132E substitution mutation in OmpK35. As these 12 included the ceftazidime-sensitive isolate A922 and only 4 of these were cefepime-resistant, the presence of this mutation within 12 isolates is inconclusive. An identical suite of 15 substitution and insertion mutations were found in the OmpK37 porin for six of the 13 sequenced isolates, while another six of 14 isolates contained a single amino acid substitution in the OmpK37 porin (Supplementary Table S1). While these mutations were observed, no clear correlation in phenotypic resistance to either ceftazidime or cefepime could be drawn. Further, while the mutations in OmpK35 and 37 appear novel and unreported in the literature, many of the mutations in OmpK36 are reported and associated with only ceftazidime resistance [80]. The presence of these porin mutations may therefore be contributing to ceftazidime and/or cefepime resistance but this requires more research to be conclusive.

As RND efflux pumps are associated with a broad substrate range and are an archetypal resistance determinant within *K. pneumoniae*, these pumps and regulatory genes were also assessed for mutations [36,82,83]. To this end, the RND efflux pump components AcrA and AcrB, as well as the repressor for the system, AcrR, and the global regulator, MarR, were also screened, with no mutations found. Further, efflux pump inhibition using efflux pump inhibitor PAβN was performed and demonstrated no change in MIC values (data not shown). Whilst overexpression of these components may occur by other mechanisms, such as transient, induced up-regulation, these data demonstrate that no mutations in the repressors could be responsible for an overexpression of the AcrAB efflux pump within these isolates.

### 3.6. The Majority of the K. pneumoniae Complex Isolates were the MDR-Outbreak-Causing Sequence Type 323

The sequenced isolates from this study spanned three different sequence types: ST4726, ST323 and ST661. The majority were of ST323 (n = 7), an ST previously associated with hospitals rather than RACFs [55,84] and recognized as an MDR clonal type [85]. Disease outbreaks have been caused by ST323 *K. pneumoniae* isolates in Africa and Australia [55,86].

Five of the sequenced isolates were ST661, an ST which has been recorded during outbreaks and found to be typically carrying carbapenemases [87,88]. Whilst none of the ST661 isolates here produced carbapenemases, all were ceftazidime-resistant, and three were additionally resistant to cefepime.

One isolate, A031, was the previously undescribed ST4726.

### 3.7. Clonal Spread of K. pneumoniae within RACFs was Observed within Facility 1

Single nucleotide polymorphism (SNP) analysis was conducted between isolates of the same sequence type to determine clonality and assess the potential for clonal spread in Facility 1 [47,48,49]. SNP analysis was performed on ST323 isolates using ST323 isolate A409 as reference (Supplementary Table S2), and ST661 isolates were assessed based on ST661 isolate A922 (Supplementary Table S3). The range of SNPs across the ST323 isolates ranged from 8 to 40 and that of the ST661 isolates ranged from 15 to 37. Despite the number of SNPs showing minor variances across the isolates spanning the two STs, the maximum number of SNPs observed was still low enough to infer clonal relatedness across all isolates of the same ST.

Of the ST323 isolates, all of which were clones, the first to be identified was isolate 2404, yielded by a faecal sample from resident 61 in January 2019. Isolate 2362 was then identified in a wastewater sample from December 2019, before isolate 2401 was detected in a faecal sample from a different resident, resident 60, in the same facility in January 2020. A413 and A409 were then recovered from the sink within the bathroom of resident 60 in Facility 1 in September 2020, while isolate A629 was recovered from a faecal sample of resident 77B of Facility 1 in December 2020. The observed clonality of isolates belonging to ST323, despite originating from different sample types and different dates, demonstrates clonal spread within Facility 1. Further, genomic assessment revealed isolate 2401, the most recently isolated, to be carrying different resistance genes, and plasmids. Together, these findings indicate the persistence and spread of clones, and potential adaptation and accumulation of resistance determinants over time. Despite their clonality, and the identification of identical resistance determinants and plasmids (except for isolate 2401 which had different plasmids than the other clones), the resistance profile of these varied. For example, a difference in cefepime MIC was observed, with isolate A409 having a cefepime MIC of >64 mg/L, compared to A413 at 1 mg/L. Conversely, isolates 2404, 2401 and A413 were all sensitive to cefepime (MIC = 1 mg/L). Whilst the ST323 clones were all ciprofloxacin-resistant, isolate A413 had a much lower ciprofloxacin MIC of 4 mg/L, while the other clones had MICs up to >64 mg/L. These differences in antimicrobial susceptibility across these clones, which could not be attributed to resistance mechanisms identified here, point to the persistence and presence of unknown resistance determinants within the RACFs.

Of the ST661 isolates, isolate A922 was the first identified and was from a wastewater sample in December 2019. Isolates 2410 and 2418 were then isolated from faecal samples of resident 50 in Facility 1, followed by isolate A095 in a faecal sample from the same resident at a later date. Lastly, a room swab collected in the rooms of participant 70 yielded isolate A529. Intra-site dissemination was therefore observed for ST661 isolates, including colonization of a resident and establishing persistence within the rooms of the facility. While small differences in antimicrobial susceptibility were observed across these isolates, these were within 1- to 2-fold differences and were not considered to be significant.

## 4. Discussion

Species of the *Klebsiella pneumoniae* complex are opportunistic pathogens which can cause serious infections and are increasingly resistant to antimicrobials [89]. The impact on mortality and the AMR capacity of *K. pneumoniae* exemplify the problem of antimicrobial resistance and mandate surveillance efforts. Current *K. pneumoniae* surveillance for outbreaks focusses on sepsis within healthcare settings like hospitals [90,91,92,93] rather than in aged care facilities. However, RACFs are now firmly implicated in the development and spread of AMR organisms [16,24,25,26,27,94], yet there is a dearth of data on the occurrence of the priority pathogen MDR *K. pneumoniae* complex and resistome burden from RACFs. Therefore, the purpose of this study was to look at the prevalence and antimicrobial susceptibility profile of the *K. pneumoniae* complex in RACFs.

A total of 147 isolates were isolated in this study from three RACFs and one retirement village in Adelaide, Australia. The three RACFs were managed by the same care provider, one that operates multiple RACFs Australia-wide, and they were thereby broadly representative RACFs. The facilities provided almost identical amenities to their residents, and the cohorts of residents (age, duration of stay) were comparable. Each of the facilities selected offered a diverse range of residency options, which included permanent care (including end of life), transitional (between hospital and community), respite (between community and RACF) and transfer (between one RACF and another). The levels of care at each of the RACFs included respite, long-term and end-of-life care. Each facility assessed in this study provided the same type of care to their residents. Potential demographic variables (socio-economic status, sex) may have been present; however, this study focussed on the prevalence of AMR pathogens within these environments and did not seek to measure these variables. To account for this and allow standardisation of comparisons, percentages, rather than total numbers, were the focus of the data presented here.

In this study, we were concerned about the incidence and spread of ESBL-producing and ciprofloxacin-resistant *K. pneumoniae* in RACFs. The incidence of carriage of putative ESBL-producing *K. pneumoniae* of 7% was in good correlation with the incidence of ESBL-producing Enterobacterales in RACFs detected in another study in Australia [57]. However, in contrast to those Enterobacterales, genomic analysis of the ceftazidime-resistant isolates from our study revealed that these organisms did not carry ESBL genes. The precise mechanism of ceftazidime resistance could not be definitively assigned, and the relative high incidence of ceftazidime resistance was most probably the result of clonal spread of resistant isolates within a facility.

Single amino acid substitution mutations were observed in outer membrane porins OmpK35, OmpK36 and OmpK37 (Supplementary Table S1). While both novel and previously reported mutations were observed, these did not correlate with a specific resistance phenotype. Further, cefepime resistance was observed in 4 of the 13 sequenced isolates, in the absence of canonical cefepime resistance determinants. With cefepime increasingly relevant as a treatment option in the context of AMR infections, an elucidation of this resistance determinant is important. Whilst this study cannot draw causal relationships, this does provide a potential further avenue for exploration into porin mutations and ceftazidime resistance and shows a need for further exploration into cefepime resistance.

Similarly, the high incidence of phenotypic ciprofloxacin resistance cannot be accounted for solely by the selection for ciprofloxacin resistance as the majority of the ciprofloxacin-resistant isolates were from plates without ciprofloxacin selection. However, genomic analysis of a subset of ciprofloxacin-resistant isolates revealed yet again clonal spread of resistant isolates carrying the *qnrB4* gene throughout the facility. Carriage of the *qnrB4* gene correlated with the carriage of beta-lactamase genes and genes for resistance to trimethoprim-sulfamethoxazole. Hence, both selective plating and intra-facility spread contributed to the relatively high incidence of ciprofloxacin resistance in *K. pneumoniae* isolates observed in this study. In comparison, ciprofloxacin resistance in *K. pneumoniae* isolates from Australian sepsis surveillance programs is currently estimated to be 7.75% [5]. Greece, recognized as having the highest levels of ciprofloxacin resistance globally from sepsis cases, reports resistance prevalence at 66.9% [18]. Data on asymptomatic *K. pneumoniae* carriage from faecal samples reported ciprofloxacin resistance at 9% in Norway [95] and 18% from community settings in Taiwan [96], while a 33% incidence of pre-admission carriage of ciprofloxacin-resistant *K. pneumoniae* was reported for neonatal hospitals in Madagascar [97].

While data are lacking on ciprofloxacin-resistant *K. pneumoniae* from RACFs specifically, other AMR Enterobacterales, such as *Escherichia coli*, are often identified from residents in aged care homes or from older patients and display ciprofloxacin resistance [96,98,99]. This co-presence could allow for intra-species dissemination of plasmid-mediated quinolone resistance genes associated with low-level plasmid resistance, such as *qnrA, qnrB* and *qnrS* [100,101,102]. Mutations in key ciprofloxacin resistance genes, such as the *parC* mutations identified in this study, have been increasingly identified in ciprofloxacin-resistant *K. pneumoniae* isolates with a range of MIC values [103,104] and this study adds to this growing body of evidence.

A review of the medical records of the participants during the previous year revealed that all the residents had received antibiotics. Although ceftazidime and cefepime were not prescribed to residents in the RACFs, the first-generation cephalosporin cephalexin was frequently prescribed and could have contributed to the development of resistance to third- and fourth-generation cephalosporins. Alternatively, the records did not capture prescribing during hospital stays and it is possible that these antibiotics could have been administered then. Ciprofloxacin or norfloxacin were among the top 10 most frequently prescribed antibiotics among the participants during the sampling period.

Given the antimicrobial resistance observed here to first- and second-line treatment options, such as cephalosporins and ciprofloxacin, resistance to last-resort and salvage antimicrobials like colistin is particularly relevant. Colistin-resistant *K. pneumoniae* typically harbour the mobile colistin-resistance gene *mcr* or a variant thereof [105,106,107], or have mutations or downregulations of the Lipid A synthesis pathway [108,109]. The *mcr* gene was not identified in the colistin-resistant isolate, indicating the observed colistin resistance may be due to another factor, such as changes in Lipid A synthesis. However, the mutations that were observed in PhoQ, PmrA, PmrB and EptA did not correlate with the observed resistance profile. Taken summarily, reported mutations correlative with resistance were observed, unreported mutations correlative with sensitivity were observed, and these findings act as contributions to the surveillance on resistance and mutations, but cannot conclusively link mutations to resistance at this stage.

Another avenue requiring further exploration is the classification of clonality, defined by the number of SNPs across core genomes. Numerous studies have used SNP analysis to define clonality, with a varying number of SNPs used as the threshold between clonality and just species-level relatedness [47,48,49]. A study comparing SNPs from *K. pneumoniae* isolates from handwashing sinks found 21 SNPs between isolates and classified these as clones [49]. Other studies of *K. pneumoniae* clonality have ranged from classifying isolates with 6 SNPs up to 75 SNPs as clones [110,111,112,113]. In our study, the highest numbers of SNPs observed between isolates classified as clones were within the range supported by the literature and isolates of the same ST were therefore classified as clones. Accordingly, we observed clonal spread of MDR *K. pneumoniae* within Facility 1.

Isolates representative of various sequence types were identified, with ST323 and ST661 shown to be the prevalent sequence types. In both Australia and worldwide, ST323 is associated with healthcare settings [55,84] and is recognized as an MDR sequence type [85]. This is consistent with our data, as all ST323 isolates herein displayed an MDR phenotype and were isolated from RACFs. Whilst our sampling was focused on asymptomatic carriage of *K. pneumoniae*, disease outbreaks caused by ST323 *K. pneumoniae* isolates have been identified in Africa and Australia [55,86]. Specifically, the ST323 outbreak in the Australian report was within a geriatric care ward [55]. With the ST323 clones identified here spreading across three residents and colonizing sinks, these data demonstrate that MDR *K. pneumoniae* can persist and disseminate within these environments, and that they represent an ongoing threat to health [114]. This is particularly troubling in an environment such as an RACF, housing vulnerable residents particularly susceptible to opportunistic pathogens. RACFs may, therefore, represent potential outbreak sites for AMR isolates warranting continued and increased surveillance. The presence of these ST323 isolates within an Australian RACF, all of which were ceftazidime-resistant, may therefore pose a real threat to the elderly residents.

ST661 isolates have been reported in healthcare settings in the UK and aquatic environments in South America, and on both occasions they were characterized by widespread dissemination throughout the localized environment [87,88]. The ST661 isolates identified here displayed MDR phenotypes; therefore, the observed persistence within the environment and outward spread between residents, coupled with the known ability of this ST to become disseminated widely over time, is therefore a clear risk factor for disease outbreak and would be an avenue for continued research of isolates within this sequence type. No reported instances of the other sequenced isolates for which a sequence type was identified, ST4726, ST997 or ST603, were found in the literature. However, given that these are STs which are now identified as correlating to isolation from RACFs and with AMR phenotypes, surveillance for these STs may be warranted. Here, clonal dissemination of *K. pneumoniae* was observed from intestinal carriage to the colonization of sinks, and other residents, consistent with other findings [49,115,116]. Crucially, the colonization of sinks by *K. pneumoniae* isolates has been repeatedly traced back as the cause of outbreaks and is therefore a risk factor for future outbreaks [49,115,116]. Sinks can act as reservoirs allowing for the persistence of pathogenic organisms like *K. pneumoniae*, with exposure causing repeated infections and potentiating outbreaks. The high potential risk of a *K. pneumoniae* outbreak within an RACF, as demonstrated by the persistence within environments and clonal spread across residents in this study, underscores the importance of continued AMR surveillance within environments housing vulnerable, immunocompromised persons such as RACFs.

## 5. Conclusions

This pilot study represents the first surveillance of AMR *K. pneumoniae* isolates within RACFs, and the first quantification of the resistome of these isolates. We found that 53% of the *K. pneumoniae* complex isolates from RACFs assessed in this study were multidrug resistant. Genomic analyses failed to conclusively identify genetic resistance determinants for the observed resistance to cefepime and colistin. Further, we identified cross-resident clonal spread and the persistence of these AMR isolates within bathroom sinks in the RACFs—representing a significant outbreak threat. These findings were consistent with a growing trend of AMR *K. pneumoniae* isolates with unclear mechanisms of resistance, and they highlight the need for further studies to elucidate these resistance determinants and for continued surveillance in this space.

## Figures and Tables

**Table 1:** NCBI database accession numbers for isolates within this study.

Accession:	Isolate:	Accession:	Isolate:
SAMN33942927	A031	SAMN33942935	2401
SAMN33942928	A026	SAMN33942936	2404
SAMN33942929	A095	SAMN33942937	2410
SAMN33942930	A529	SAMN33942938	2418
SAMN33942931	A629	SAMN33942939	A922
SAMN33942932	A413	SAMN33942940	2362
SAMN33942933	A409		

**Table 2:** The distribution of the 147 K. pneumoniae complex isolates by facility and sample type.

		Sample Type			
	*K. pneumoniae* Complex Isolates n (%)	Faecal n (%)	Urine n (%)	Facility Swab n (%)	Wastewater n (%)
**Facility 1**	97 (66)	46 (47)	0 (0)	4 (4)	47 (48)
**Facility 2**	25 (17)	16 (64)	0 (0)	0 (0)	9 (36)
**Facility 3**	7 (5)	5 (71)	2 (29)	0 (0)	0 (0)
**Retirement**	18 (12)	15 (83)	0 (0)	0 (0)	3 (17)
**Total:**	147 (100)	82 (56)	2 (1)	4 (2)	59 (40)

**Table 3:** MIC distribution (mg/L) for 147 *K. pneumoniae* complex isolates recovered from three RACFs and one retirement living cohort.

	MIC (mg/L)												
Antimicrobial	<0.125	0.125	0.25	0.5	1	2	4	8	16	32	64	>64	% Resistant
Ceftazidime	3	1	5	19	23	18	11	4	0	4	2	57	46
Cefepime	32	3	12	33	21	15	8	3	1	3	1	15	16
Ciprofloxacin	13	5	8	19	9	17	26	24	7	4	7	8	69
Tobramycin	0	0	6	43	62	21	3	1	3	4	3	1	10
Amikacin	0	0	3	4	15	72	36	9	6	1	0	1	5
Meropenem	31	0	6	13	35	46	14	2	0	0	0	0	0
Colistin	0	0	3	11	41	90	1	0	0	1	0	0	1
	**<0.125**	**0.125**	**0.25**	**0.5**	**1**	**2**	**4**	**8**	**>8**				
Trimethoprim sulfamethoxazole	2	8	16	14	13	16	9	7	62				47

Vertical black lines indicate EUCAST breakpoint values, with values lower than breakpoint considered as ‘susceptible’, and above considered as ‘resistant’. Green shading is indicative of the number of isolates resistant to each compound.

**Table 4:** MIC distribution (mg/L) for 147 *K. pneumoniae* complex isolates for three commonly used biocides.

MIC (mg/L)														
Antimicrobial		≤0.125	0.125	0.25	0.5	1	2	4	8	16	32	64	>64	% Tolerant
**Biocide**	CHG	2	0	1	0	5	8	33	41	32	21	2	2	1
	TRI	5	1	7	14	20	33	27	26	5	0	1	8	46
	BZK	0	0	0	0	4	2	0	1	21	88	27	4	21

Vertical black lines indicate reported ECOFF values [33], with values lower than breakpoint considered as ‘susceptible’, and above considered as ‘tolerant’. Green shading is indicative of the number of isolates tolerant to each compound. CHG: chlorhexidine digluconate; TRI: triclosan; BZK: benzalkonium chloride.

**Table 5:** EUCAST breakpoints, sequence types (STs) and minimum inhibitory concentrations (MICs) for antimicrobials used in this study and sample type information of sequenced *K. pneumoniae* complex isolates.

MIC (mg/L)										
			CTZ	FEP	CIP	TOB	AMI	MER	COL	SXT
	EUCAST Breakpoint		>4	>4	>0.5	>2	>8	>8	>2	>4
Isolate	ST	Source								
A031	4726	Faecal	2	0.125	0.25	0.25	0.25	<0.125	1	4
A922	661	Wastewater	32	8	2	1	1	1	2	>8
A026	323	Faecal	64	1	2	1	1	4	2	>8
A095	661	Faecal	>64	32	4	2	2	1	2	>8
A409	323	Facility	>64	1	2	0.25	0.25	2	1	>8
A413	323	Facility	64	1	4	1	1	2	2	>8
A529	661	Facility	32	0.5	1	1	1	1	2	>8
A629	323	Faecal	64	1	4	0.5	0.5	1	2	>8
2362	323	Wastewater	>64	0.5	4	0.5	0.5	2	2	>8
2401	323	Faecal	64	1	4	0.5	0.5	2	2	>8
2404	323	Faecal	64	1	4	1	1	4	2	>8
2410	661	Faecal	>64	64	8	0.25	0.25	<0.125	32	>8
2418	661	Faecal	>64	64	8	1	1	1	2	>8

CTZ: ceftazidime; FEP: cefepime; CIP: ciprofloxacin; TOB: tobramycin; AMI: amikacin; MER: meropenem; COL: colistin; SXT: trimethoprim-sulfamethoxazole.

## Data Availability

The collective WGSs of isolates used in this study were deposited to the NCBI database BioProject ‘PRJNA949397’ (Table 1).

## Supplementary Materials

The following supporting information can be downloaded at: https://www.mdpi.com/article/10.3390/microorganisms12040751/s1, Supplementary Table S1: Mutations in genes known to confer antimicrobial resistance, with isolate A031 as reference, Supplementary Table S2: Matrix of SNP pair counts for ST323 isolates, with A409 used as reference, Supplementary Table S3: Matrix of SNP pair counts for ST661 isolates, with A922 used as a reference.
