# Supplementary material for: Resistome Analysis of Klebsiella pneumoniae Complex from Residential Aged Care Facilities Demonstrates Intra-facility Clonal Spread of Multidrug-Resistant Isolates
Source: Microorganisms. 2024 Apr 8;12(4):751. doi: 10.3390/microorganisms12040751 (PMC11051875; doi:10.3390/microorganisms12040751)
Supplement: Supplementary file 1 [file microorganisms-12-00751-s001.zip › Blaikie_etal_Supplementary_Table S1.pdf]

Supplementary Table S1: Mutations in genes known to confer antimicrobial resistance, with isolate A031 as reference.

	**A031**	**A026**	**A095**	**A529**	**A629**	**A413**	**A409**	**2401**	**2404**	**2410**	**2418**	**A922**	**2362**
	Ciprofloxacin phenotype R (resistant) or S (sensitive)												
	R	R	R	R	R	R	R	R	R	R	R	R	R
**GyrA**	X	X	X	X	X	X	X	X	X	X	X	X	X
**GyrB**	X	X	X	X	X	X	X	X	X	X	X	X	X
**ParC**	X	N304S	X	X	N304S	N304S	N304S	N304S	X	X	X	X	N304S
**ParE**	X	X	X	X	X	X	X	X	X	X	X	X	X
	Colistin phenotype R (resistant) or S (sensitive)												
	S	S	S	S	S	S	S	S	S	R	S	S	S
**PhoP**	X	X	X	X	X	X	X	X	X	X	X	X	X
**PhoQ**	X	X	X	X	X	X	X	X	X	X	X	X	X
**PmrA**	M66I	X	M66I	M66I	X	X	X	X	X	M66I	M66I	M66I	X
**PmrB**	X	T246A	T240M T246A	T240M T246A	T246A	T246A	T246A	T246A	T246A	T240M T246A	T240M T246A	T240M T246A	T246A
**EptA**	T149M T224M K399I	X	T224M	T224M	X	X	X	X	X	T224M	T224M	T224M	X
**EptB**	X	X	X	X	X	X	X	X	X	X	X	X	X
**MgrB**	X	X	X	X	X	X	X	X	X	X	X	X	X
	Ceftazidime phenotype R (resistant) or S (sensitive)												
	R	R	R	R	R	R	R	R	R	R	R	S	R
	Cefepime phenotype R (resistant) or S (sensitive)												
	S	S	R	S	S	S	R	S	S	R	R	S	S

**OmpK35**	x	K132E	K132E	K132E	K132E	K132E	K132E	K132E	K132E	K132E	K132E	K132E	K132E
**OmpK36**	x	x	L184INS<br>G191T<br>F200Y<br>H220N<br>N204L<br>S228INS<br>R229K<br>D231A<br>K232L<br>I312L<br>L320I<br>E349D<br>D351S<br>R355H<br>R356N	L184INS<br>G191T<br>F200Y<br>H220N<br>N204L<br>S228INS<br>R229K<br>D231A<br>K232L<br>I312L<br>L320I<br>E349D<br>D351S<br>R355H<br>R356N	x	x	x	L184INS<br>G191T<br>F200Y<br>H220N<br>N204L<br>S228INS<br>R229K<br>D231A<br>K232L<br>I312L<br>L320I<br>E349D<br>D351S<br>R355H<br>R356N	x	L184INS<br>G191T<br>F200Y<br>H220N<br>N204L<br>S228INS<br>R229K<br>D231A<br>K232L<br>I312L<br>L320I<br>E349D<br>D351S<br>R355H<br>R356N	L184INS<br>G191T<br>F200Y<br>H220N<br>N204L<br>S228INS<br>R229K<br>D231A<br>K232L<br>I312L<br>L320I<br>E349D<br>D351S<br>R355H<br>R356N	L184INS<br>G191T<br>F200Y<br>H220N<br>N204L<br>S228INS<br>R229K<br>D231A<br>K232L<br>I312L<br>L320I<br>E349D<br>D351S<br>R355H<br>R356N	x
**OmpK37**	x	I184M	X	X	I184M	I184M	I184M	X	I184M	X	X	X	I184M
