# Supplementary material for: Resistome Analysis of Klebsiella pneumoniae Complex from Residential Aged Care Facilities Demonstrates Intra-facility Clonal Spread of Multidrug-Resistant Isolates
Source: Microorganisms. 2024 Apr 8;12(4):751. doi: 10.3390/microorganisms12040751 (PMC11051875; doi:10.3390/microorganisms12040751)
Supplement: Supplementary file 1 [file microorganisms-12-00751-s001.zip › Blaikie_etal_Supplementary_Table S2.pdf]

Supplementary Table S2: Matrix of SNP pair counts for ST323 isolates, with A409 used as reference.

Sample Information				SNP Matrix						
Location	Sample ID	Sample type	Collection date		2362	A409	A413	A629	2401	2404
Facility 1	N/A	Wastewater	02/12/2019	2362	0	40	28	35	22	24
Facility 1	60	Sink	30/09/2020	A409	40	0	24	41	24	24
Facility 1	60	Sink	30/09/2020	A413	28	24	0	33	14	16
Facility 1	77B	Faecal	09/12/2020	A629	35	41	33	0	23	25
Facility 1	60	Faecal	24/01/2020	2401	22	24	14	23	0	8
Facility 1	61	Faecal	17/01/2019	2404	24	24	16	25	8	0
