# Supplementary material for: Resistome Analysis of Klebsiella pneumoniae Complex from Residential Aged Care Facilities Demonstrates Intra-facility Clonal Spread of Multidrug-Resistant Isolates
Source: Microorganisms. 2024 Apr 8;12(4):751. doi: 10.3390/microorganisms12040751 (PMC11051875; doi:10.3390/microorganisms12040751)
Supplement: Supplementary file 1 [file microorganisms-12-00751-s001.zip › Blaikie_etal_Supplementary_Table S3.pdf]

Supplementary Table S3: Matrix of SNP pair counts for ST661 isolates, with A922 used as a reference.

Sample Information				SNP matrix					
Location	Sample ID	Sample type	Collection date		A922	2410	2418	A095	A529
Facility 1	N/A	Wastewater	02/12/2019	A922	0	22	29	26	34
Facility 1	50	Faecal	04/12/2019	2410	22	0	15	32	24
Facility 1	50	Faecal	04/12/2019	2418	29	15	0	33	35
Facility 1	50B	Faecal	14/07/2020	A095	26	32	33	0	40
Facility 1	70	Room Swab	20/10/2020	A529	34	24	35	40	0
